# Global incidence of Necrotizing Enterocolitis: a systematic review and Meta-analysis

**DOI:** 10.1186/s12887-020-02231-5

**Published:** 2020-07-13

**Authors:** Amer Alsaied, Nazmul Islam, Lukman Thalib

**Affiliations:** 1 Department of Public Health, College of Health Sciences, QU Health, Qatar University, Doha, Qatar; 2 HMC Medical Cooperation, Doha, Qatar; 3 Sidra Medicine, Doha, Qatar

**Keywords:** Necrotizing Enterocolitis, Incidence, Systematic review, Meta-analysis

## Abstract

**Background:**

Necrotizing Enterocolitis (NEC) is a major cause of morbidity and mortality in the Neonatal Intensive Care Unit (NICU), yet the global incidence of NEC has not been systematically evaluated. We conducted a systematic review and meta-analysis of cohort studies reporting the incidence of NEC in infants with Very Low Birth Weight (VLBW).

**Methods:**

The databases searched included PubMed, MEDLINE, the Cochrane Library, EMBASE and grey literature. Eligible studies were cohort or population-based studies of newborns including registry data reporting incidence of NEC. Incidence were pooled using Random Effect Models (REM), in the presence of substantial heterogeneity. Additional, bias adjusted Quality Effect Models (QEM) were used to get sensitivity estimates. Subgroup analysis and meta-regression were used to explore the sources of heterogeneity. Funnel plots as appropriate for ratio measures were used to assess publication bias.

**Results:**

A systematic and comprehensive search of databases identified 27 cohort studies reporting the incidence of NEC. The number of neonate included in these studies was 574,692. Of this 39,965 developed NEC. There were substantial heterogeneity between studies (I^2^ = 100%). The pooled estimate of NEC based on REM was 7.0% (95% CI: 6.0–8.0%). QEM based estimate (6.0%; 95% CI: 4.0–9.0%) were also similar. Funnel plots showed no evidence of publication bias. Although, NEC estimates are similar across various regions, some variation between high and low income countries were noted. Meta regression findings showed a statistically significant increase of NEC over time, quantified by the publication year.

**Conclusion:**

Seven out of 100 of all VLBW infants in NICU are likely to develop NEC. However, there were considerable heterogeneity between studies. High quality studies assessing incidence of NEC along with associated risk factors are warranted.

## Background

Last three decades have witnessed great improvements in the neonatal intensive care, in particular, with the introduction of surfactant therapy and the subsequent improvement in the care of respiratory distress syndrome (RDS) that reduced the mortality among preterm newborns [1]. With better survival of premature babies, Necrotizing Enterocolitis (NEC) became more common and its burden became more prominent [2].

Multiple population-based studies, some based on large cohort studies, have reported the incidence of NEC to vary from 2 to 13% in preterm and Very Low Birth Weight (VLBW) infants [2–6]. The variation in the incidence were attributed to differences in the risk factor profiles as well as differing population at risk, detection rate and inclusion and exclusion criteria. There is no pooled estimate of the incidence of NEC worldwide. Furthermore, there is no incidence data from some regions such as North Africa, the Middle East or the Arab Gulf region, apart from a single study from the UAE [7].

With the continuing improvement in survival of preterm newborns, the modifiable risk factors of NEC need to be studies and made use of in developing appropriate interventions to reduce the incidence and impact of NEC. In this context, clinicians and researchers have attempted to identify the factors associated with risk and prognosis of NEC. It was reported as early as the 1980’s, that there exist an association between rapid advancement of feeding and the onset of NEC [8]. Subsequent reports showed preterm birth [9, 10], small birth weight [9–11] and race [11] were also to be important risk factors. Contemporary reports confirm these initial reports and expand the list to include a few more. More recent studies have shown that preterm birth [3, 12]. low birth weight [2, 12], rapid advancement of feeding, race and ethnicity, use of glucocorticosteriods [2], maternal infection [13], indomethacin therapy [14], congenital pneumonia [14], meconium aspiration [15], asphyxia [15], blood transfusion [15] and hypotension within the first week of life [16] are also potential contributing factors.

This study aims to systematically review the incidence reported from different parts of the world to synthesize a global incidence of confirmed NEC in VLBW infants. The study also aims to explore the regional variability as well as other potential factors that can explain variability in the incidence.

## Methods

The recommendations from the Preferred Reporting Items for Systematic Review and Meta-Analysis (PRISMA) served as the guide in collating and reporting this review [17].

### Eligibility criteria

Eligible studies included cohort or population-based studies of newborns including registry data. Both prospective and retrospective studies were included. Studies reporting the number, frequency or incidence of confirmed NEC in preterm infants or VLBW infants along with appropriate denominator were included. Studies that reported data on subgroups of infants with specific exposures such as congenital heart disease, perinatal infections, preterm rupture of membrane, or sepsis were excluded when the incidence could not be extracted. Studies with unclear case definitions of NEC were also excluded. Randomized controlled trials had strict selection criteria therefore including them would have caused selection bias and reduced the external validity of our pooled estimate. Hence, experimental studies that were assessing the effect of an intervention on a selected group of neonates were excluded. Case series where there were no denominator data to compute the incidence were also excluded.

Incidence is used as opposed to prevalence because of the natural history of NEC and its short duration of disease. It is envisaged that findings form this study would provide clinically important baseline data as the starting point for studies that aim to reduce the incidence of NEC.

### Population and outcome

The VLBW infants formed the population of this study and the outcome of was the incidence of NEC stage II or above according to Bells criteria.

### Search data bases

The database search was started in September 2018 and last updated in December 2019. The databases searched were PUBMED, MEDLINE (Ovid), EMBASE, the Cochrane Library. Additional databases searched included: African Index Medicus Database, Latin America and Caribbean Center of Health Science International, Open Grey, IndMED, KoreaMED, Virtual Health Library, National Library of Australia and Social Care Online.. Further manual search included looking for relevant studies in the reference lists of the included papers.

### Search strategy

The search strategy was developed by the authors to include a comprehensive database search using broader search terms such as: “Enterocolitis, Necrotizing”, “Epidemiology”, “Incidence”, “Cohort Studies”, and “population-Based studies”, “cohort studies”, “epidemiological data”, “prematurity”, “Very low birth weight”, “clinical study”, “cohort analysis”, and “‘human”. Additional MeSH (Medical Subject Heading) term based search complemented the above search. When appropriate using the above terms with a combination of ‘and’ and ‘or’ in accordance with search engine specifications were carried out. The search string used for PUBMED is given in Supplementary file S1 as an illustration.

### Study selection

Two review authors (AA and NI) independently assessed the titles and abstracts of all citations retrieved by the search for relevance against the inclusion criteria. Then the full-text versions of studies considered potentially eligible were retrieved. The same two authors independently assessed the full papers for eligibility, with disagreements resolved through input of the third author. The duplicate records and those not eligible were eliminated and a PRISMA flow chart was created to depict the study selection process.

### Data extraction

Data form the eligible studies were extracted and collated on to data tables. Name of the authors, year of publication, data on the time period covered by the study, location of the study, inclusion and exclusion criteria of the study (Table 1), the reported population at risk and whether it was VLBW infants or preterm infants, case definition, incidence or number on NEC cases and size of population at risk (Table 2) were collected. The data extraction process was performed by AA and checked by NI. Any discrepancies is resolved by discussion.

**Table 1:** Characteristics of the included studies

Author/year	data base studied	Inclusion criteria	Exclusion criteria	Population at risk reported	NEC case definition	Comment on VLBW	Incidence (cumulative)
Stoll et al. 2010 [18]	NICHD	VLBW infants born in NRN centers GA 22–28 wks.	Congenital anomalies	preterm infants among a VLBW pool	clinically	exclusively VLBW infants	11%
Llanos et al. 2002 [3]	Finger Lakes regional center	all live births in an area of 6 counties. Data obtained from a state-wide registry.	not clear	all newborns in the regional center were accounted for but specific report on NEC stage II and above among the VLBW infants is extracted	NEC stage II and above	population based study but reported specific parameters on VLBW	3.29%
Luig et al. 2005 [4]	New South Wales – state-wide data base. NICUS Neonatal Intensive Care Unit Study	population based study - all preterm infant s between 24 and 28 wks.	not clear	all preterm infants 24–28 weeks of gestation	Clinical definition as confirmed NEC on a set of criteria similar to Bell’s criteria	the mean birth weight and SD of the three epochs were 959 (240), 946 (204), and 935 (240)	7.67%
Holman et al. 2006 [19]	data from discharge registry (the kid’s Inpatient Database) compiled data from 27 states, 2700 hospitals accounting for 10% uncomplicated births from these hospitals	the data is a comprehensive cohort of 10% of all live births in the specified hospitals.	NE after 1 month of age	VLBW infants	ICD 9 -CM code NEC 777.5	Specific report NEC and VLBW infants is presented exclusively VLBW infants	4.34%
Youn 2015 [16]	Korean Neonatal Network. Admissions into 55 participating neonatal intensive care unites	all live births or admissions within 28 days. VLBW infants. Data collected	52 were diagnosed with NEC II and Spontaneous bowel perforation and were excluded	VLBW infants	bell’s stage II and above	exclusively VLBW infants	6.41%
Qian et al. 2017	95 major referral centers in 29 provinces. Representative of NICU care in the areas	all LBW infants were included.	not specified	the study reports specific parameters of VLBW infants	bell’s stage II and above	reports on VLBW infants are extracted from the publications	2.53%
Ahle et al. 2013 [12]	Swedish National Board of Health and Welfare, the National Patient Register, the Swedish Medical Birth Register and the National Cause of Death Register	all newborns between 1987 and 2009	incomplete identity number	VLBW infants	ICD 9 or ICD 10 code 777F or P77	reported all birth weights. Exact parameters of each weights group are available too	2.68%
Wojkowska-Mach et al. 2014	Polish Neonatal Surveillance Network	all VLBW infants born in PNSS	missing records	VLBW infants	NEC defined according to Gastmeier’s (clinical)	exclusively VLBW	8.68%
Boo et al. 2012 [14]	Malaysian National Neonatal Registry includes NICUs in Malaysia	All VLBW infants in the MNNR.	excluded infants less than 501 g	VLBW infants	bell’s stage II and above	exclusively VLBW infants	6.20%
Wong et al. 2013	Population based study: New South Wales and Australian Capital Territory NICUs included in the NICUS	Low birth weight infants	congenital malformation, syndromes with neurodevelopmental disorders, death in the labor room	low birth weights infants	Bell’s staging criteria	the population was of low birth weights (mean birth weight in two groups was 895 and 917 g.	7.81%
Fanaroff 2003 [20]	NICHD. Retrospective data analysis was performed to compare three epochs.	Registry data	not specified	VLBW infants	not clear	VLBW infants	6.23%
Chedid et al. 2008	Single large Neonatal tertiary referral center	all admission to a single tertiary center in Alain between 2004 and 2006	life threatening malformation, died in labor room, less than 500 g	VLBW infants (exclude less than 500 g	not clear, pneumatosis intestinal or perforation was used a confirmation	all are VLBW	5.78%
Agrawel et al. 2015	data from single largest tertiary hospital in Singapore. Viability threshold less than 25 wks. Gestation	Neonates from High risk VLBW data base with GA < 29 wks.	still birth and miscarriage, less than 23 weeks of gestation	VLBW and pre-term	bell’s stage II and above	exclusively VLBW infants	6.98%
Patole et al. 2016 [21]	single center experience. Comprehensive retrospective cohort comparing a before and after intervention	all neonates less than 34 weeks of gestation within a 2-year period before and after intervention	neonates involved in a clinical trial for the same purpose	the study reported all neonates less than 34 wks. But data on < 28 weeks and epoch 1 were extracted	bell’s stage II and above	the birth weight of the preterm babies was not specifically reported	6.40%
Verstreate et al. 2016	Retrospective cohort study from a single e center using a local audit data base	All neonates in the hospital system	neonates with culture samples that had probably contamination	data on VLBW was extracted only	clinical definition	the data extracted represents exclusively VLBW infants	16.23%
Harkin et al. 2017	Finish Medical Birth Register (preterm < 32 wks.) 22–31. all VLGA 4143	all born less than 32 weeks of gestation	congenital malformations sever chromosomal defects or death before 7 days od life	less than 28 weeks of gestation	clinical criteria	50% less than 1000 g in the entire populations. But weight of the < 28 weeks of gestation was not specified	6.58%
Andersen et al. 2018	birth cohort of the California Office Statewide Health and Development (OSHPD)	all live births with GA 22–36	chromosomal abnormalities	GA less than 28 weeks	ICD-9	no clear specification of the birth weight of the preterm subpopulation	9.10%
Suciu et al. 2017 [22]	From three Romanian hospitals (tertiary centers) data from two different periods 2007–2010 and 2011–2014	all preterm babies less than 28 weeks of gestation	chromosomal abnormalities and birth defects or missing data	preterm babies less than 28 weeks of gestation	bell’s stage II and above	the mean birth and SD of the two epochs were 809 +/− 211 and 958 +/− 149	17.08%
Patel et al. 2016	Prospective 0bservational multicenter birth cohort study evaluating VLBW infants from multiple Level III neonatal centers for exposure blood transfusion (a risk of NEC)	VLBW infants	not specified	VLBW infants	bell’s stage II and above. Cumulative incidence at 8 weeks	exclusively VLBW infants	7.34%
Bajwa et al. 2011 [23]	Swiss Neonatal Network. Double verification by the Swiss Society of Neonatology.	The data set includes all infants < 32 weeks of gestation and > 23 wks.	infants who died in labor room	preterm less than 28 weeks of gestation	clinical definition	no comment on the birth weight of the subpopulation less than 28 weeks of gestation	4.95%
Narang et al. 1993 [24]	Single Neonatal Intensive Care Unit	All live births during the period January 1986 to September 1990	Not reported	VLBW infants and pretenn infants of gestational age less than 32 weeks	modified Bell’s criteria	Majority are VLBW infants	1.5%
Lodha 2019 [25]	Tertiary neonatal intensive care units participating in the Canadian Neonatal Network	born at 22 to 28 weeks’ gestational age	birth outside a tertiary-level NICU, moribund at birth, designated as needing palliative care before delivery, had major congenital anomalies, or lacked cord clamping information	22 to 28 weeks’ gestational age	According to the modified Bell criteria, and NEC stage 2 or higher was classified as medical or surgical.	No estimate of the percentage of VLBW infants	9%
Boghossian 2018 [26]	Vermont Oxford Network center	Inborn, singleton infants without congenital malformations	Infants with unknown sex and missing or implausible birth weight	Infants of gestational ages 22 to 29 weeks	diagnosed at surgery or postmortem or required at least 1 clinical sign (eg, bilious gastric aspirate, abdominal distension, or occult blood in stool) and at least 1 radiographic finding (eg, pneumatosis intestinalis, hepatobiliary gas, or pneumoperitoneum).	the mean birth weight and SD of the each weeks reported.	9%
Persson 2018 [27]	7 national networks in high-income countries that are part of the International Neonatal Network for Evaluating Outcomes in Neonates	All singleton infants born alive in high-income countries who were very preterm (24-31 weeks’ gestation) and with a birth weight of less than 1500 g	Multiple pregnancies and major congenital malformations	Very Preterm and Very Low-Birth-Weight Infants	Necrotizing enterocolitis was analyzed in a subgroup of the cohort because data from the UKNC were not available for stage 2 or 3 NEC	Very Preterm and Very Low-Birth-Weight Infants	3%
Suzuki 2018 [28]	Neonatal Research Network	Extremly preterm infants born between 2008 and 2012	Infants who died within 6 days, infants with congenital anomalies, whose sex was undetermined, or whose records were missing data	extremely preterm infants	NEC was defined as stage II/III cases, according to the classifications of Bell	All are VLBW with extremly preterm	4%
Boghossian 2018 [29]	852 US centers participating in the Vermont Oxford Network	Infants born between 154 days (22 weeks and 0 days) and 209 days (29 weeks and 6 days) of gestation	Multiples and infants born with congenital malformations	Large for Gestational Age Infants	NEC was diagnosed at surgery or postmortem or required at least 1 clinical sign (eg, bilious gastric aspirate, abdominal distension, occult blood in stool) and at least 1 radiographic finding (eg, pneumatosis intestinalis, hepatobiliary gas, or pneumoperitoneum)	Mean and SD birth weights reported	7%
Beltempo 2018	Canadian Neonatal Network	Infants born from 22 to 28 weeks’ GA and admitted to 30 Level 3 neonatal intensive care units (NICUs)	Infants moribund on admission or where palliative care was provided at birth due to imminent mortality, infants with major congenital anomalies, and infants with missing SNAP-II	Extremely preterm infants	NEC is defined as stage ≥2 according to Bell’s criteria	Mean and SD birth weights of both cohort is reported	8%

**Table 2:** Summary of the 27 studies included in the quantitative analysis

Period	Author/Year	Location	Population at risk	Cases of NEC in population at risk	Population at risk	Incidence
2003–2007	Stoll et al. 2010 [18]	US	VLBW infants	**not reported**^a^	**9575**	**11.0%**
1991–1998	Llanos et al. 2002 [3]	US	VLBW infants	**47**	**1425**	**3.29%**
86/87, 92/93, and 98/99	Luig et al. 2005 [4]	Australia	Extremely premature	**127**	**1655**	**7.67%**
2000	Holman et al. 2006 [19]	US- 27 states	VLBW infants	**2554**	**58,810**	**4.34%**
201–2014	Youn 2015 [16]	Korea	VLBW infants	**149**	**2326**	**6.41%**
2011	Qian et al. 2017	China	VLBW infants	**221**	**8727**	**2.53%**
1987–2009	Ahle et al. 2013 [12]	Sweden	VLBW infants	**473**	**17,608**	**2.68%**
2009	Wojkowska-Mach et al. 2014	Poland	VLBW infants	**79**	**910**	**8.68%**
2007	Boo et al. 2012 [14]	Malaysia	VLBW infants	**222**	**3601**	**6.20%**
1998–2004	Wong et al. 2013	Australia	VLBW infants	**199**	**2549**	**7.81%**
87/88, 93/94,99/2000	Fanaroff 2003 [20]	US	VLBW infants	**786**	**12,628**	**6.23%**
2004–2006	Chedid et al. 2008	UAE	VLBW infants	**10**	**173**	**5.78%**
2000–20,209	Agrawel et al. 2015	Singapore	VLBW infants	**50**	**835**	**6.98%**
2008–2010	Patole et al. 2016 [21]	Australia	Extremely premature	**16**	**250**	**6.40%**
2002–2011	Verstreate et al. 2016	Belgium	VLBW infants	**158**	**973**	**16.23%**
2005–2013	Harkin et al. 2017	Finland	Extremely premature	**170**	**1025**	**6.58%**
2007–2012	Andersen et al. 2018	US-California	Extremely premature	**1360**	**14,941**	**9.10%**
2007–2010	Suciu et al. 2017 [22]	Romania	Extremely premature	**82**	**480**	**17.08%**
2010–2014	Patel et al. 2016	US-Atlanta	VLBW infants	**44**	**598**	**7.34%**
2000–2004	Bajwa et al. 2011 [23]	Switzerland	Extremely premature	**64**	**1283**	**4.95%**
1986–1990	Narang et al. 1993 [24]	India	VLBW infants			
2011–2015	Lodha 2019 [25]	Canada	Extremely premature	**412**	**4680**	**9%**
2006–2016	Boghossian 2018 [26]	United States	VLBW and Extremely premature	**18,129**	**194,736**	**9%**
2007–2015	Persson 2018 [27]	Sweden	Extremely premature	**2077**	**76,360**	**3%**
2008–2012	Suzuki 2018 [28]	Japan	Extremely premature	**296**	**8245**	**4%**
2006–2014	Boghossian 2018 [29]	USA	Extremely premature	**10,376**	**138,869**	**7%**
2010–2015	Beltempo 2018	Canada	Extremely premature	**778**	**9230**	**8%**

^a^ The number of NEC cases was calculated from the incidence and the baseline population for this study

### Risk of Bias assessment

**Fig. 1:**
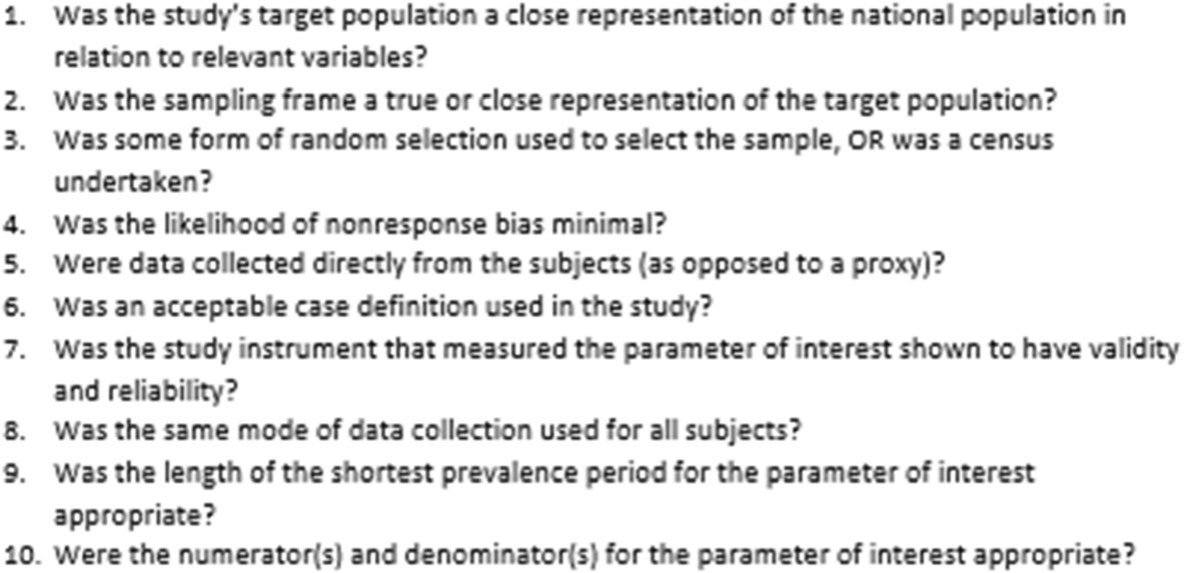
The 10 criteria used to assess the risk of bias in each included studies

All the included studies were assessed for internal and external validity using the criteria put forward by Hoy et al. that were specific for prevalence and incidence studies (Fig. 1). This tool was developed based on key domains they identified to be important in assessing the risk of bias in incidence and prevalence studies. The tool was subsequently validated and found to have good validity [30].

### Data synthesis

Pooling the incidence estimates was done after arcsine transformations of the data as it has been shown to stabilize variance and reduce bias [31]. Heterogeneity was assessed using the Cochrane Q test and Higgin’s I^2^ value. Smaller *p* values and I^2^ > 50% were indicative of significant heterogeneity [32, 33]. As Cochrane guidelines suggest use of Random Effect Models (REM) when significant heterogeneity is encountered [34] we employed REM models estimates to arrive at the main conclusion. Further, bias adjusted Quality Effect Models (QEM) [35] were used to obtain sensitivity estimates to check the robustness of the REM estimates. Quality scores obtained using Hoy’s criteria were used in fitting the QEM.

Forest plots were used to display the incidence of NEC with corresponding 95% confidence intervals. We used Hunter plots to assess the publication bias as Hunter et al. have shown the classical funnel plot to be in-appropriate for proportion studies such as prevalence or incidence [36].

A-priori planned meta-regression was performed to evaluate if the publication year has any impact on the variability of the incidence and as a possible cause of heterogeneity. This was also thought to be important to understand if the long term trend in incidence of NEC to see if they are on a rise or decline. Further subgroup analysis by region based on income category of the countries provided by World Bank and population at risk (VLBW or extremely premature) was also carried out [37]. This sub-group analysis was not an a-priori decision but an attempt to explain the variability in NEC due to substantial heterogeneity. Groups consisted of high income countries (HIC) and low middle-income countries (LMIC).

The meta analyses were carried out using MetaXL [31] and the subgroup analysis and meta regression were carried out using Comprehensive Meta-Analysis (CMA-V3) software [38].

## Results

### Study characteristics

**Fig. 2:**
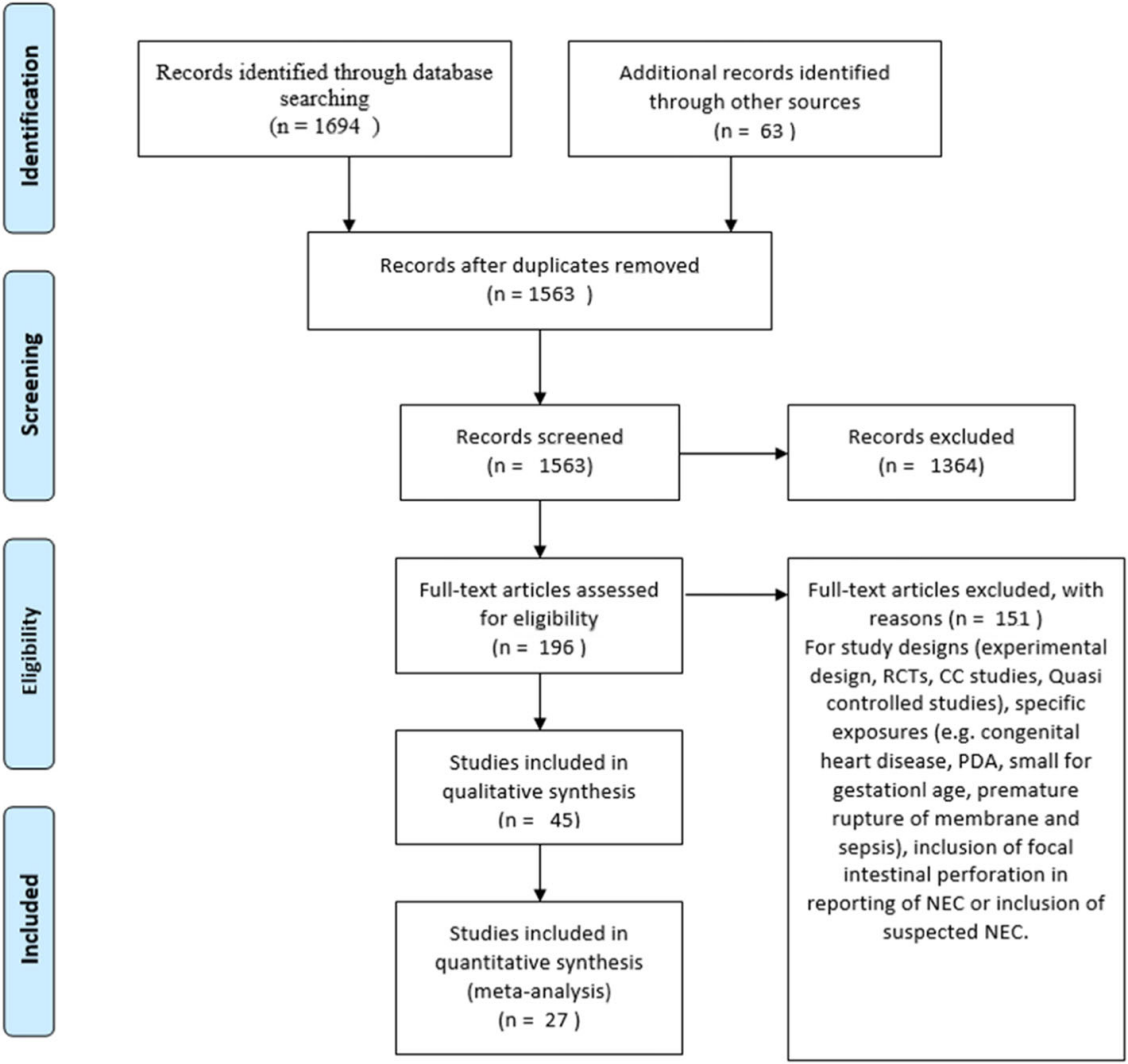
Flow chart depicting the studies screened, selected and included based on PRISMA

The total number of publications identified for screening was 1694. The process of selection of eligible studies are depicted as a PRISMA flow chart (Fig. 2). A total of 27 studies were found to fulfill the eligibility criteria and included in the review (Table 1). The number of neonate included in these studies was 574,692. Of these, 39,965 neonates developed confirmed NEC (Table 2). The studies covered a broader geographical areas globally. Some regions had multiple studies other areas had none. A total of eight studies were reported from the United States covering a number of states including: California, Texas, Atlanta, Connecticut, and New York [3, 6, 9, 18, 19, 39–41]. Multiple studies were also reported from the Europe including Poland, Romania, Finland, Belgium, Sweden and Switzerland [12, 13, 23, 39, 42, 43]. Also, four studies were done in China, Korea, Singapore and Malaysia [14, 16, 44, 45]. Three studies from Australia [4, 21, 46], one from the Middle East [7] and one from India [24].

The publication year of the studies ranged from 1988 to 2019, but the majority were carried out after 2000. Some of the studies focused on evaluating a certain exposure [7, 9, 21, 43], however, the data presented in these papers were not limited to the exposure groups and data from the general population was extracted to compute the incidence (Table 2).

### Qualitative review

Andersone et al. reviewed a cohort data from the California Office Statewide Health Planning And Development [OS HPD] [39]. Upon retrograde calculation of the number of NEC cases and dividing them by a total number of NICU preterm babies the incidence of NEC was 9.1%. Whilst, Patole et al. conducted a retrospective cohort study reviewing 1755 neonates who were less than 34 weeks of gestation [21]. The aim was to study the effect on the incidence of NEC. In the control group (prior to the initiation of probiotic), there were 835 babies. Among those 250 were preterm with gestational age less than 28 weeks. Stage II or above NEC was found in 16 cases (6% of preterm controls).

Stoll et al. [38] analyzed data on 9575 newborns with very low birthweight and extremely low gestational age. The incidence in this population was 11%. Llanos et al. [3] reported the incidence among VLBW infants therefore was 3.29%. They used a retrospectively conducted a population-based survey from six counties in New York State. Holeman et al. analyzed the hospital discharge data from the Kid’s Inpatient Database from the year 2000 [47]. Among those born with weight less than 1500 g, the number of cases was 2554 and the rate was 4342.8 per 100,000 live births annually with an incidence of 4.3%. Fanaroff et al. evaluated VLBW infants and compared three periods of time: 1987–1988, 1993–1994, and 1999–2000 [20]. The analysis aimed to compare the outcome across the time periods. They showed that the incidence of NEC did not change over time.

Bajwa et al. reviewed the data from the Swiss neonatal network that conatins comprehensive population-based data of all infants in Switzerland [23]. The analysis included 368,055 infants born between 2000 and 2004, Ahle et al. collected data from the Swedish National Board of Health and Welfare, the National Patient Register, the Swedish Medical Birth Register and The National Cause of Death Register between 1987 and 2009 [12]. The incidence of NEC in less than 750 g, 750–999 g, 1000–1499 g and 1500–2499 g were 5.31, 4.16, 1.52, and 0.007%, respectively.

Verstrate et al. based on a retrospective cohort of 5134 neonatal intensive care unit admissions from a single hospital Belgium found 973 cases were born with a very low birthweight of less than 1500 g [42]. The incidence of NEC with stage II or above, in this subgroup was 16.23%. Härkin et al. reviewed the data from the national Registry of preterm infants born between 2005 and 2013 in Finland [43]. The incidence of NEC among preterm babies was therefore 16.58%. Wójkowska-Mach et al. reviewed the Polish Neonatal Surveillance Network for all VLBW infants recorded in the national registry. They used clinical criteria for the definition of NEC and 79 of 910 babies developed NEC [13].

Suciu et al. reviewed data from three tertiary centers in Romania. The study included 480 preterm babies born before 28 weeks of gestation [22]. The incidence was estimated to be 16.6%. The Bell’s criteria were used to define cauterizing enterocolitis as stage II and above in this study. Agarwal et al. collected data from the single largest neonatal center in Singapore with a vitality threshold defined at 25 weeks of gestation [45]. The database included all neonates who are with VLBW and gestational age less than 29 weeks. Bell’s classification was used to define NEC. 50 babies among 835 developed NEC.

Qian et al. reported data extracted retrospectively from 95 major referral centers and hospitals in china covering a large area of 29 provinces [44]. VLBW infants were specified and the incidence of NEC according to Bell’s criteria was presented in 2011. The data included 46,686 infants of whom, 8727 were born with VLBW. The incidence of confirmed NEC in VLBW infants was 6.5 among a cohort of 8727 infants.

Youn et al. reported a large cohort from South Korea. Among a total of 2326 infant with VLBW, 145 (6.8%) were diagnosed with confirmed NEC stage II of above [16]. Boo et al. collected data retrospectively from 31 neonatal intensive care units around Malaysia on NEC defined by Bell’s criteria among VLBW infants. Among the 3601 babies included, 222 developed NEC. Of these 197 had NEC II and 25 were NEC III or above according to Bell’s staging criteria. The incidence was 6.2% [14]. Luig et al. reported data on all infants born between 24 to 28 weeks of gestation in New South Wales and England, over three different time periods: 1986–1987, 1992–1993, and 1998–1999 [4]. The population included 1655 cases from the three groups divided to 360, 622, and 673 cases in time periods 1986–1987, 1992–1993, and 1998–1999 respectively. Over the entire population the incidence was 7.67%.

Wong et al. conducted a retrospective cohort study reviewing 2549 neonates from 10 neonatal intensive care units serving New South Wales in Australia [46]. This study population accounted for all preterm infants in the region of Australia between 1998 and 2004. The conducted the analysis complaining those exposed to steroids and those who were not. The incidence of NEC was 7.8% as 199 cases developed necrotizing enterocolitis among 2549 preterm babies born before 29 weeks of gestation.

Narang et al. 1993, collected 2200 admissions to the NICU during the period January 1986to September 1990 [24]. Among them 33 developed NEC (Bell’s stage ≥2). The incidence was 1.5%. Chedid et al. reviewed 173 newborns from 1 Tertiary Referral Center in UAE, Al Ain. All the cohort were born with weight less than 1500 g [very low birthweight infants] [7]. NEC was diagnosed clinically. Among the study population, 10 babies developed confirmed NEC. The incidence of NEC was 5.8%.

Lodha et al. 2019, compared neonatal outcomes after deferred cord clamping and immediate cord clamping in extremely low-gestational-age neonates from tertiary neonatal intensive care units participating in theestimated incidence based on Canadian Neonatal Network in 2019 was 9% (43)9%.

Boghossaan et al. 2018, examined infants of gestational ages 22 to 29 weeks born between January 2006 and December 2016 at a Vermont Oxford Network center in the United States were. NEC developed in 18,129 among the 194,736 infants. The incidence of NEC was 9% [26]. Persson et al. 2018, conducted a retrospective cohort study at 7 national networks in high-income countries that are part of the International Neonatal Network for Evaluating Outcomes in Neonates and used prospectively collected data on 76,360 very preterm, singleton infants. 2077 infants developed NEC and the incidence was 3% [27].

Suzuki et al. 2018, retrospectively examined 8245 extremely preterm infants born between 2008 and 2012 using Neonatal Research Network database in Japan. They estimated incidence to be 4% [28]. Boghossian et al. 2018, collected 138,869 large for gestational age infant’s data from 852 US centers participating in the Vermont Oxford Network. The incidence of NEC was 7% (10,376 new cases) [29]. Beltempo et al. 2018, collected data about extremely preterm infants born from 22 to 28 weeks’ gestational age Canadian Neonatal Network. Study population was 9230 among them 778 developed NEC. The incidence of NEC was 8% [48].

### Assessment of risk of Bias

**Fig. 3:**
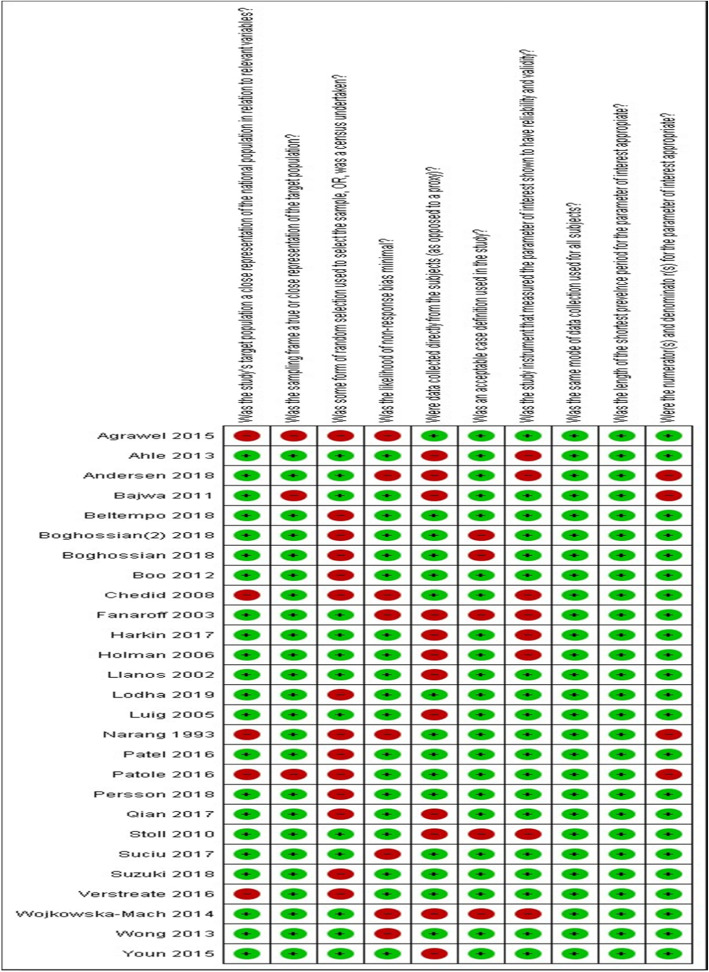
Risk of bias plot that shows the methodological quality assessment of the 27 studies included

The quality assessment of 27 individual studies carried out as per Hoy et al. [30] criteria are summarized graphically presented in Fig. 3. Studies performed very highly on components like use of consistent mode of data collection from all infants as well as sufficient follow up time required for the desired outcome to occur. However, only about 50% of the studies had a random selection of samples. Overall, most studies scored high and 17 out of 27 studies had a lower risk of bias based on a cut of 8/10 or more as suggested by the Hoy’s criteria.

### Quantitative analysis of incidence

**Fig. 4:**
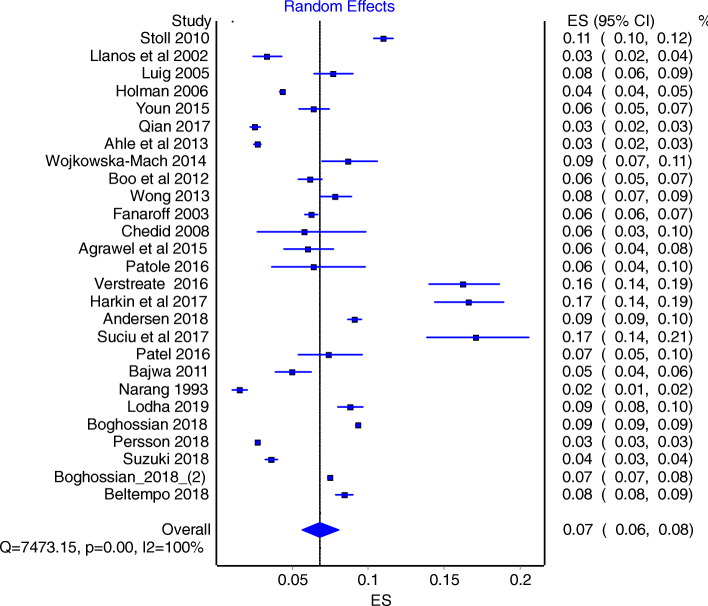
Forrest plot obtained using Random Effect Model

**Fig. 5:**
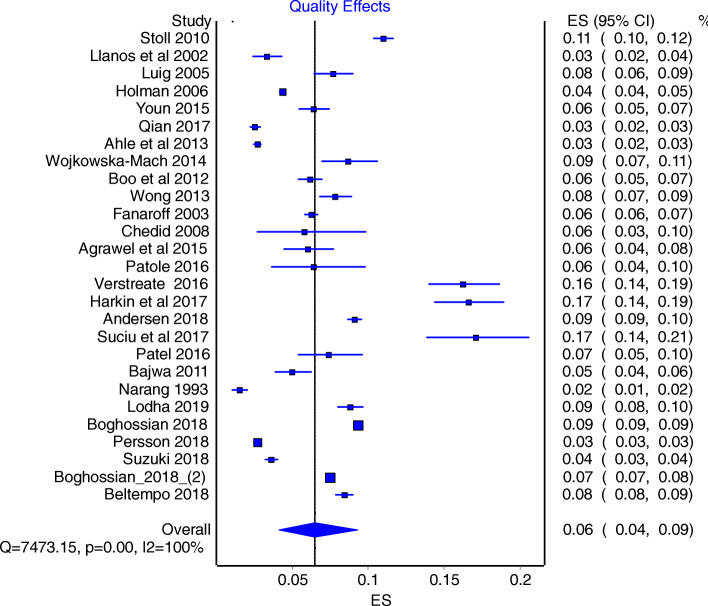
Forrest plot obtained using Quality Effect Model

There were significant heterogeneity between studies, as indicated by I^2^ value of 100% and the Cochrane Q- statistics (value =7473; *P* < 0.0001). As such we used REM as the main model to obtain our conclusions. REM estimate were 7.0% (95% CI: 6.0–8.0%) (Fig. 4), and additional quality adjusted QEM provided a sensitivity estimate of 6.0% (95% CI: 4.0–9.0%) (Fig. 5).

### Publication bias

**Fig. 6:**
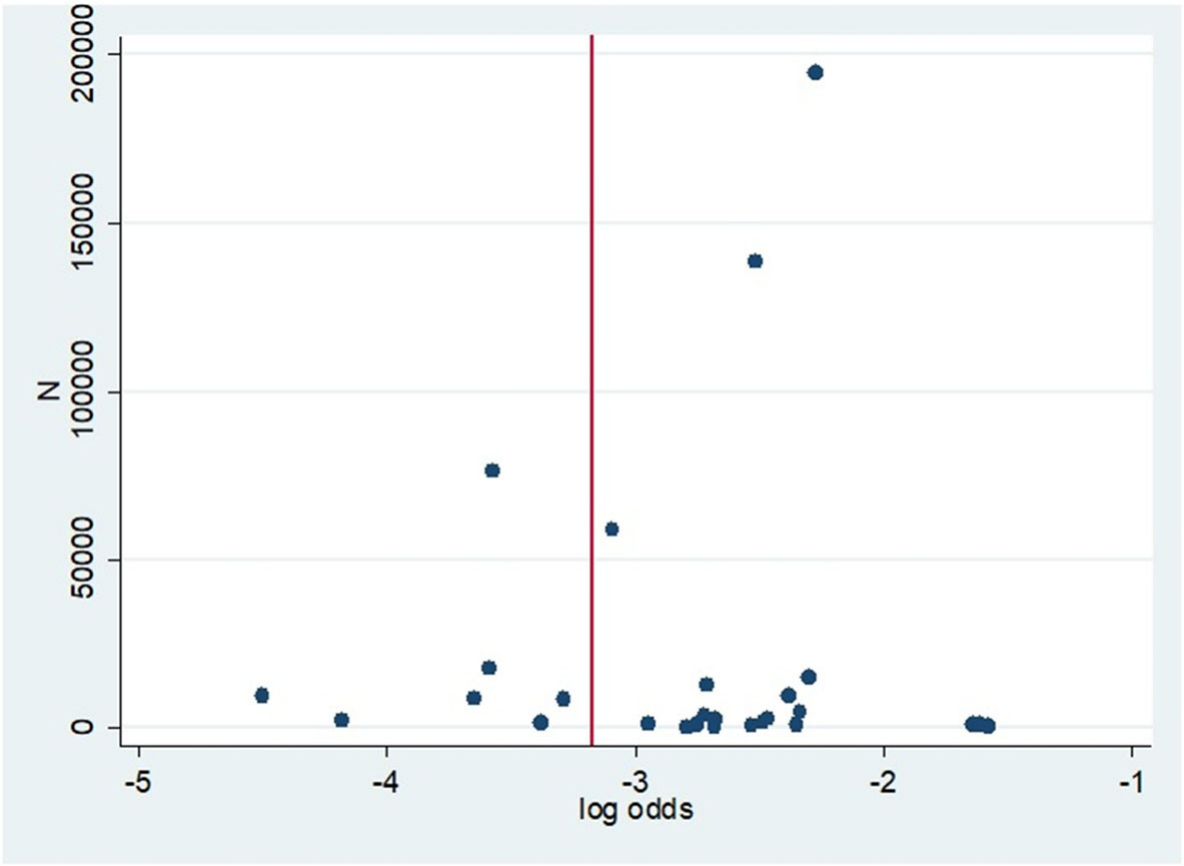
Hunter’s plot used to assess the publication bias

Hunter’s modified funnel Plot [36] as appropriate for the incidence data used to evaluate the publication bias appear to not to show a serious concern (Fig. 6). Further, the Eggers regression confirmed that publication bias was not statistically significant (two tailed *p-value* = 0.80). The Kendall’s Tau test statistics was also not statistically indicating less likely that these studies encountered publication bias (two tailed *p-value* = 0.936).

### Subgroup analysis

There was no significant regional variation between North America, Western Europe and Australia as well as Asia (Table 3). There appear to be some variation between HIC and LMIC countries, although these differences were not statistically significant. No significant variation between VLBW infants and extreme prematurity was found.

**Table 3:** Subgroup analysis by region and income

Region	Pooled Incidence (%)	95% CI
All	6.0	[4.0, 9.0]
North America, Western Europe and Australia	4.3	[2.5, 6.6]
Asia	3.9	[1.4, 7.3]
**Income**		
All	6.0	[4.0, 9.0]
High income countries (HIC)	7.0	[4.0, 10.0]
Low and middle-income countries (LMIC)	3.0	[1.0, 6.0]
**Population at risk**		
All	6.0	[4.0, 9.0]
VLBW infants	6.0	[3.0, 9.0]
Extremely premature	7.0	[2.0, 13.0]

### Meta-regression

**Fig. 7:**
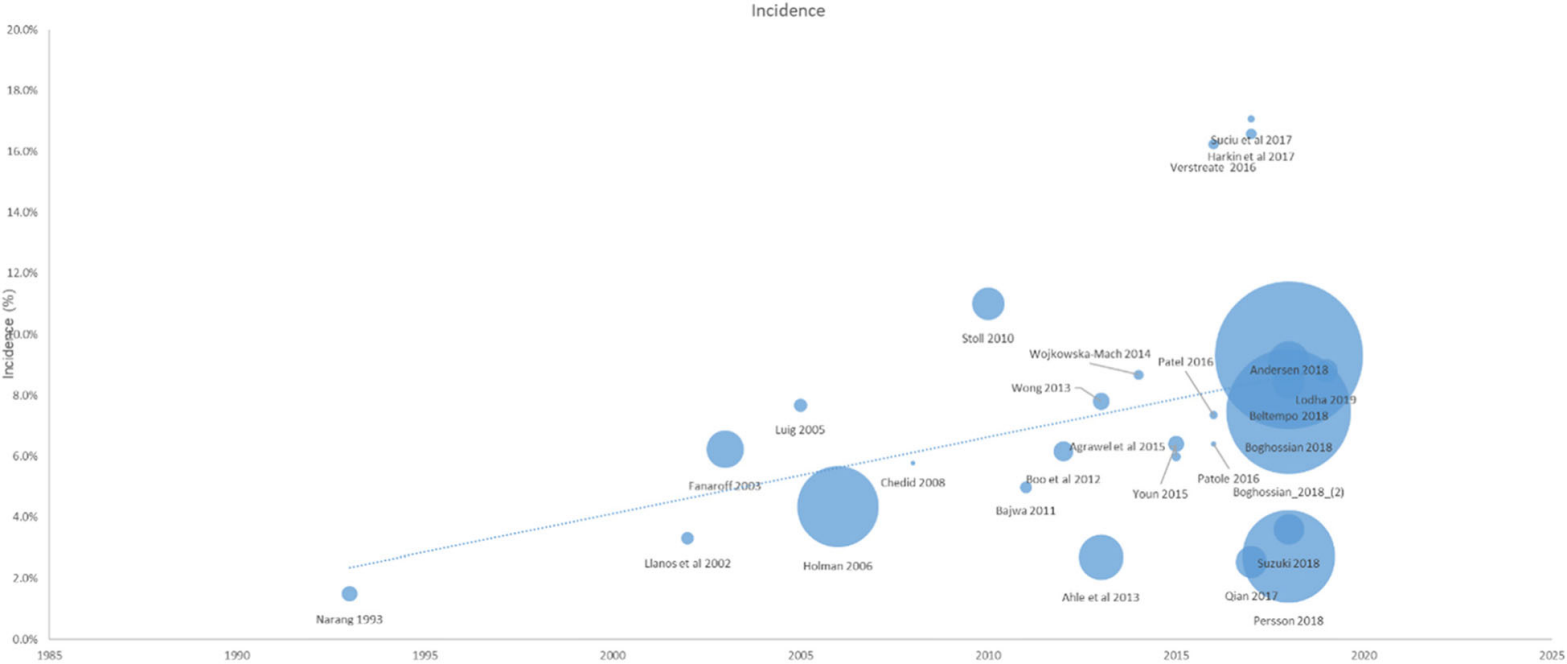
Meta regression of incidence over time

There was a statistically significant increase in the log event rate over time, quantified by the publication year (Fig. 7).

## Discussion

This study is perhaps the first attempt to provide a pooled estimate of the incidence of Necrotizing Enterocolitis in VLBW infants. Seven out of 100 of all VLBW infants in NICU are likely to develop NEC as per our synthesis. However, there were considerable heterogeneity in the estimates across studies. Such important variability may be driven by myriad of factors including the variation in the quality of health care systems.

Subgroup analysis based on geographic regions did not reveal any differences (i.e. South East Asia versus Europe, North America and Australia). However, when countries reporting the data on NEC were re-classified based on income levels using Word Bank classifications the incidence in high income counties (HIC) varied from the low and mid income countries (LMIC), although, these differences were not statistically significant. Such variation may be attributed to the fewer published studies from LMIC and potential under power to detect any differences. However, it is also possible that slightly lower incidence reported in LMIC may be due to higher gestational age cutoff point for resuscitation used in case of extreme prematurity. It is also possible that the sicker babies in LMIC may have had higher risk of mortality. As a result the population of neonates in LMIC may appear healthier and at lesser risk of developing NEC.

The increase in the incidence of NEC over time that our study found using meta-regression maybe attributed to multiple factors. Improvement in neonatal care and better survival of premature infants are possible causes as well as improvement in diagnosis and reporting. Increase in incidence of NEC over time can also be attributed to lack of wide scale prevention strategies. Ahle et al. demonstrated a j-shaped distribution of incidence over time in Sweden. While the incidence was 150 per 10,000 live births among VLBW infants in the late 80s, it increased to approximately 800 per 10,000 live births in VLBW, a multiple fold increase in later decade [12]. This increase may be related to variations in local health services. However, findings from the analysis of the NICHD data base from the United States reported [20] showed a different picture. They reviewed VLBW infants from three epochs: 1987–1988, 1993–1994, and 1999–2000. Their analysis compared the incidence across these three periods and they demonstrated that the incidence of NEC did not change over time. The data presented in our analysis represents a wider time period and a set of more diverse healthcare settings. Due to paucity of data available from lower income countries, the pooled estimate may have limited external validity and not fully generalizable to all global settings and populations.

Our findings, however, should be understood in the light of some limitations that this study encountered. Only 12 out 26 studies could be considered to be of higher quality and this may be linked to the substantially heterogeneity that we encountered. Although, we employed quality effect models to adjust for variation in study qualities, substantial heterogeneity noted in this study does pose a threat to evidence synthesis. The diagnosis of NEC using Bell’s criteria or similar definitions schemes is a day to day clinical challenge. To a certain extent, two clinicians may justifiably disagree on labeling a baby as confirmed NEC versus suspected NEC.

## Conclusions

Seven out of 100 infants admitted to NICU and are VLBW are likely to develop NEC. However, there are substantial variability in incidence reported from different parts of the world, likely be due to differences in clinical and health settings in addition to methodological variations. Larger and higher quality studies on incidence of NEC and associated factors, particularly form low and middle income countries are warranted.

## Supplementary information

**Additional file 1.**

## Data Availability

Input data for the analyses are available from the corresponding author on request.

## Abbreviations

FEM: Fixed effect model
ICD: International classification of diseases
MeSH: Medical subject heading
NEC: Necrotizing enterocolitis
NICHD: National institute of child health and human development
PRISMA: Preferred reporting items for systematic review and meta-analysis
QEM: Quality effect model
REM: Random effect model
RDS: Respiratory distress syndrome
VLBW: Very low birth weight
