# Supplementary material for: Global incidence of Necrotizing Enterocolitis: a systematic review and Meta-analysis
Source: BMC Pediatr. 2020 Jul 13;20:344. doi: 10.1186/s12887-020-02231-5 (PMC7359006; doi:10.1186/s12887-020-02231-5)
Supplement: Supplementary file 1 — Additional file 1. [file 12887_2020_2231_MOESM1_ESM.docx]

**Search Strategy: Pubmed [Dec 27 2019]**

(necrotising enter*[tiab] OR necrotizing enter*[tiab] OR intestine perforat*[tiab] OR pseudomembranous colitis*[tiab] OR necrotizing enterocolitis totalis[tiab]) AND ("infant, low birth weight"[MeSH Terms] OR low birth weight infant[title/abstract] OR low birth weight infants[title/abstract] OR low[ title/abstract] OR extremely[title/abstract] OR severe[title/abstract] OR birth weight*[tiab] OR lbw[tiab] OR vlbw[tiab] OR elbw[tiab]) AND ("infant, premature"[MeSH Terms] OR premature infant[title/abstract] OR premature infants[title/abstract] OR preterm infant*[title/abstract] OR "infant, extremely premature"[MeSH Terms] OR extremely premature infant[title/abstract] OR sga[tiab] OR prematur*[tiab] OR baby[tiab] OR babies[tiab] OR nicu[tiab] OR preterm* [title/abstract]) AND (risk factor[tiab] OR risk factors[tiab] OR association[tiab] OR epidemiolog*[tiab] OR incidence*[tiab] OR prevalence*[tiab] OR morbidity[title/abstract] OR morbidities[title/abstract])

Supplementary Figure 1: Subgroup analysis by income

Supplementary Figure 2: Subgroup analysis by population at risk
